# Effects of proanthocyanidin-functionalized hydroxyapatite nanoparticles on dentin bonding

**DOI:** 10.1007/s00784-024-05836-7

**Published:** 2024-07-24

**Authors:** Tattiana Enrich-Essvein, Santiago González-López, Alejandro B. Rodríguez-Navarro, Carolina Cifuentes-Jiménez, Tatjana Maravic, Claudia Mazzitelli, Vittorio Checchi, Uros Josic, Annalisa Mazzoni, Lorenzo Breschi

**Affiliations:** 1 Department of Operative Dentistry, School of Dentistry, University of Granada, Campus de Cartuja, Colegio Maximo s/n, Granada, E-18071 Spain; 2 Department of Mineralogy and Petrology, Faculty of Sciences, University of Granada, Granada, Spain; 3 Department of Biomedical and Neuromotor Sciences (DIBINEM), University of Bologna, Bologna, Italy; 4 Department of Surgery, Medicine, Dentistry and Morphological Sciences, University of Modena and Reggio Emilia, Modena, Italy

**Keywords:** Collagen cross-linkers, Dentin biomodification, Nanomaterials, MMPs, Zymography, Microtensile bond strength

## Abstract

**Objectives:**

To evaluate the effect of proanthocyanidin-functionalized hydroxyapatite nanoparticles (nHAp_PA) used as pretreatment at different concentrations on the microtensile bond strength (µTBS) and endogenous enzymatic activity (MMPs) on pH-cycled dentin after 24 h and 6 months of artificial aging.

**Materials and methods:**

Fifty human sound dentin blocks were randomly assigned to 5 groups (*n* = 10): (i) negative control (no treatment); (ii) positive control (pH-cycling); (iii) pH-cycling + 2% nHAp_PA for 60s; (iv) pH-cycling + 6.5% nHAp_PA for 60s; (v) pH-cycling + 15% nHAp_PA for 60s. A self-etch adhesive was used for bonding procedures before resin composite build-ups. Specimens were tested with the µTBS test after 24 h and 6 months of laboratory storage. The proteolytic activity in each group was evaluated with gelatin zymography and in situ zymography. Data were statistically analyzed (*p* < 0.05).

**Results:**

At 24 h, the µTBS of the experimental groups were significantly higher than the controls (*p* ≤ 0.001), and no differences were observed between different concentrations (*p* > 0.05). Artificial aging significantly decreased bond strength in all groups (*p* ≤ 0.008); however, nHAp_PA 2% still yielded higher bonding values than controls (*p* ≤ 0.007). The groups pretreated with nHAp_PA exhibited lower MMP-9 and MMP-2 activities compared to the positive control group and almost the same enzymatic activity as the negative control group. In situ zymography showed that after 6 months of aging, nHAp_PA 2% and nHAp_PA 6,5% decreased enzymatic activity as well as the negative control.

**Conclusions:**

Dentin pretreatment with nHAp_PA increased the bonding performance of a self-etch adhesive and decreased MMP-2 and MMP-9 activities after 6 months.

## Introduction

Restorative therapy with adhesive resins and resin composits is widely used to replace decayed dental hard tissues. Although there is scientific evidence of the excellent clinical performance of composite restorations, failures still occur in resin-based dental restorations [1]. Therefore, effective and long-lasting adhesion remains the greatest challenge in adhesive dentistry. The formation of a firm and stable hybrid layer (HL) with a complete infiltration and adequate polymerization of the resin in the demineralized collagen matrix is the key factor for the retention of resin restorations [2]. In case of incomplete resin infiltration, a series of hydrolytic degradation phenomena and enzymatic degradative processes of denuded collagen fibrils mediated by host-derived proteases, such as matrix metalloproteinases (MMPs) and cysteine cathepsins (CT), can take place leading to the early loosening of restorations [3, 4].

Considering that bond longevity depends on the stability and integrity of demineralized collagen fibrils, different strategies are currently being developed to prevent degradation processes and stabilize collagen matrix. Biomodification of dental hard tissues using bioactive materials prior to the adhesive protocol is one of these promising strategies [5]. These biomaterials often possess interesting properties for dentin biomodification such as remineralizing, antiproteolytic, and crosslinking activities [1]. Among the different cross-linkers that have been investigated in the recent literature [6, 7], proanthocyanidin (PA) has demonstrated great potential in stabilizing the exposed collagen matrix by forming covalent, ionic, and hydrogen bonds and hydrophobic interactions [8, 9]. The application of PA to dentin has been shown to improve its mechanical properties [10–13], inhibit MMPs and CTs [14–16], and remineralize artificial caries lesions [17–19], while exhibiting low toxicity and high biocompatibility [12, 20].

A recent report demonstrated a significant potential of PA when functionalized with synthetic nano-hydroxyapatite (nHAp) to improve the interaction of the mineral within collagen fibrils [21]. The formation of the nHAp_PA complex promotes remineralization while improving collagen stability in demineralized dentin for a clinically accepted treatment time [21]. Nevertheless, the ability of these nanoparticles to stabilize the HL and reduce the endogenous dentinal enzymatic activity has not yet been evaluated. Therefore, the aim of this study was to evaluate the effect of proanthocyanidin-functionalized hydroxyapatite nanoparticles (nHAp_PA) used at different concentrations as a pretreatment primer on the microtensile bond strength (µTBS). Additionally, the endogenous enzymatic activity in demineralized dentin using a self-etch adhesive was evaluated after 24 h and 6 months of artificial storage. The null hypotheses tested were that (1) there are no differences in the microtensile bond strength between the experimental groups, (2) there are no differences in the MMPs activities between the experimental groups, and that (3) aging does not influence the bond strength and MMPs activity.

## Materials and methods

### Reagents and chemicals

Grape seed extract produced from *Vitis vinifera L.* (95% proanthocyanidin) was provided by Infisa - Instituto Fitológico s.l. (Barcelona, Spain, batch nº 200,938). The reagents for the synthesis of nanoparticles and production of artificial caries (i.e., AgNO_3_, CaCl_2_, NaH_2_PO_4_, acetic acid, KCl, and NH_3_) were supplied by Pancreat (Barcelona, Spain) and Scharlau (Barcelona, Spain). Ultrapure deionized water (Milli-Q Millipore, Merck, Molsheim, France) was used throughout the experiment. The reagents used for the gelatin and in situ zymography were obtained from Merck, France, unless stated otherwise. The gelatin substrate E-12,055 and Pierce Protease Inhibitor Tablets, EDTA-free were obtained from Thermo Fisher Scientific, (Wilmington, DE, USA).

### Synthesis of nanoparticles

According to previous protocols [21, 22], proanthocyanidin-functionalized hydroxyapatite nanoparticles (nHAp_PA) were synthesized by chemical precipitation method. First, 100 ml of 0.1 M calcium chloride [CaCl_2_] aqueous solution with deionized Milli-Q water was prepared. Then, grape seed extract (0.5%, m/m) was dissolved in 340 ml of deionized water and added to the CaCl_2_ solution at room temperature under stirring for 30 min. Subsequently, an aqueous solution of disodium hydrogen phosphate [Na_2_HPO_4_] (0.1 mol/L, 60 ml) was added dropwise (1 ml/min) to the solution of the chelate complex consisting of calcium ions and grape seed. The pH of the solutions was kept constant at pH 9.0 throughout the experiment by adding ammonia [NH_3_]. The suspension was stirred at 60 °C for 2 h and then aged at 37 °C for 72 h. After artificial aging, the solution was centrifuged and washed for 10 min five times with deionized water and dried for 24 h at 37 °C. To determine the particle size, morphology and crystalline phase of the nanoparticles, transmission electron microscopy (LIBRA 120 PLUS TEM; Zeiss, Germany) and X-ray diffraction using CuKα radiation (X’Pert Pro; PANalytical, The Netherlands) were employed. The average size of the nanoparticles was 15 nm. Additional data on the characteristics of the nanoparticles can be found in our previous work [21].

### Dentin specimen preparation and experimental design

**Fig. 1:**
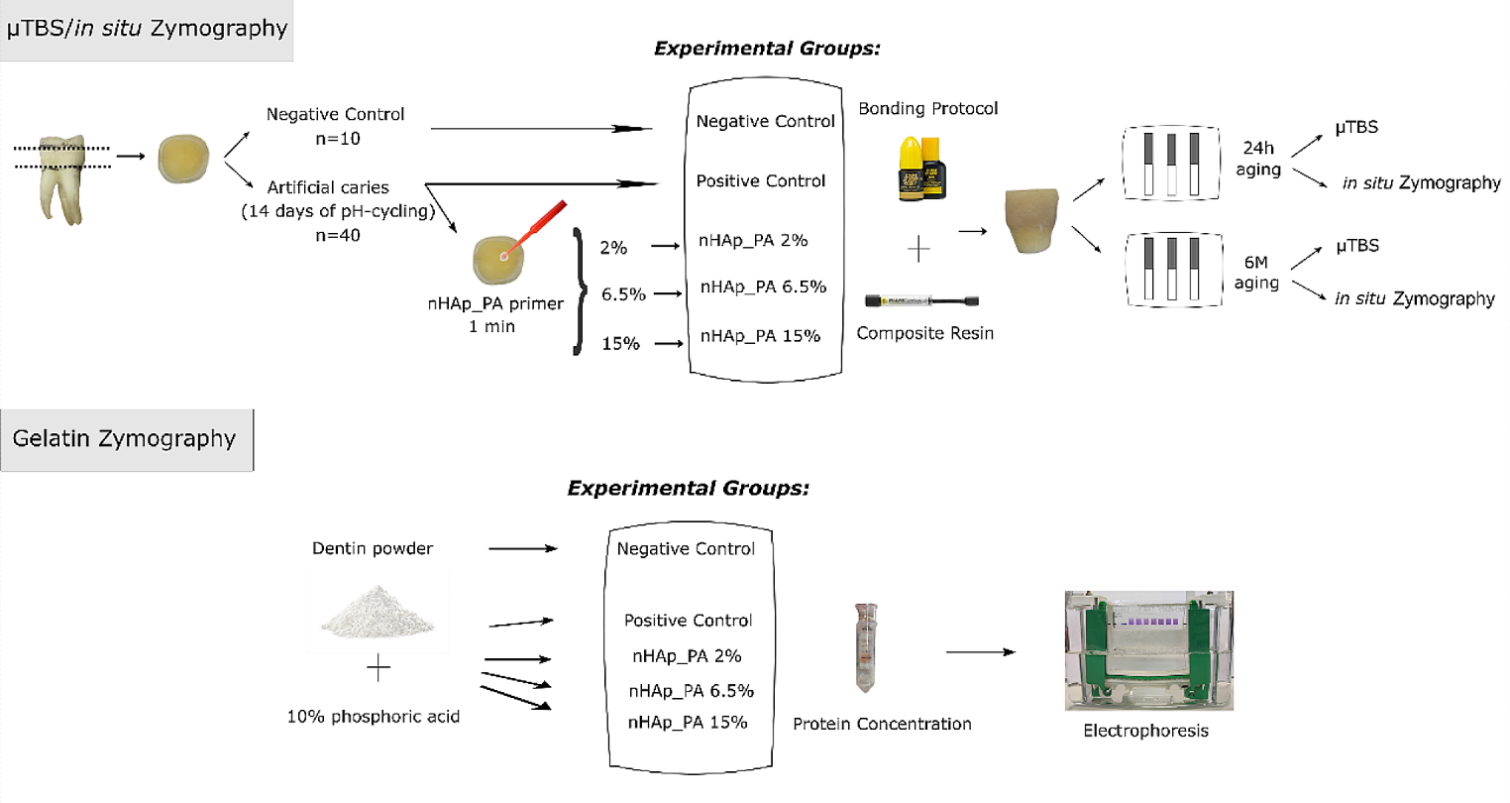
Schematic diagram of the experimental design

The current research was approved by the Ethics Committee of the University of Granada, Spain (#1896–2020). The entire experimental design is illustrated in Fig. 1. Fifty sound human permanent molars collected after informed consent were cleaned and stored in 0.1% thymol solution at 4 °C until sample preparation. The occlusal enamel and the roots 2 mm below the cementoenamel junction were removed using an Isomet 11/1180 (Buehler, Lake Bluff, USA) with a diamond cut-off wheel (MOD 13, Struers, Ballerup, Denmark). The exposed dentin surface was wet-ground with 280-grit silicon carbide paper for 5 s to create a standardized smear layer. Then, the samples were rinsed with distilled water to remove debris.

Teeth were randomly assigned into five experimental groups (*n* = 10 per group) according to the dentin treatment: (i) negative control (no treatment); (ii) positive control (14 days of pH-cycling); (iii) pH-cycling + nHAp_PA 2%; (iv) pH-cycling + nHAp_PA 6.5%; (v) pH-cycling + nHAp_PA 15%. Specimens corresponding to the negative control were immersed in distilled water. The remaining groups were subjected to an artificial caries procedure by a pH-cycling method [23]: specimens were immersed in 10 ml of a demineralizing solution (2.2 mM CaCl_2_, 2 mM NaH_2_PO_4,_ and 50 mM acetic acid adjusted to a pH of 4.8) for 8 h and, subsequently, in 10 ml of a remineralizing solution (1.5 mM CaCl_2_, 0.9 mM NaH_2_PO_4,_ and 0.15 M KCl and adjusted to a pH of 7.0) for 16 h. This process was carried out for 14 days, at 37 °C, without agitation. The solutions were renewed and pH values were monitored daily. After pH-cycling, another control group (i.e., group ii / positive control) was created. The remaining groups were subjected to pretreatment with nHAp_PA-containing solutions. The dentin pretreatment solutions were prepared at 2%, 6.5% and 15% w/v by dissolving nHAp_PA in deionized water and the final pH were 7.5, 7.35 and 7.25, respectively.

Bonding procedures.

The control groups (i.e., negative and positive) did not receive surface pretreatment prior to bonding procedures. The experimental groups were pretreated with 2%, 6.5% or 15% nHAp_PA solution for 60 s, which would be an acceptable clinical time, with gentle agitation using a fully saturated applicator. Then, the surfaces were rinsed and gently air-dried. After dentin pretreatment, all groups, including control groups, were bonded with Clearfil Se Bond 2 self-etch adhesive (Kuraray Noritake, Okayama, Japan) following the manufacturer’s instructions (Table 1). Finally, composite resin build-ups of about 6 mm height were constructed by applying 2 mm layers of Brilliant EverGlow (Coltene, Altstatten, Switzerland). Each increment layer was light-cured for 20 s using an LED curing light (Blue-phase, Ivoclar Vivadent, Schaan, Liechtenstein), which was verified to have a light output of 1200 mW/cm^2^, as indicated by the unit’s radiometer (Bluephase Meter, Ivoclar Vivadent, Schaan, Liechtenstein). Finally, the samples were stored in distilled water for 24 h. Materials used for bonding procedures are described in Table 1.

**Table 1:** Materials used in the study, chemical composition, batch number, manufacturer, and application mode

Materials	Chemical composition	Batch number	Manufacturer	Application
Grape Seed Extract	95% Proanthocyanidin	210,296	Infisa, Barcelona, Spain	2%, 6.5% or 15% w/v in aqueous solution: Apply on dentin surface for 60 s with gentle agitation using a fully saturated applicator. Then, the surfaces were rinsing and gentle air-dried.
Clearfil SE Bond 2	Primer: 10-MDP, HEMA, Hydrophilic aliphatic dimethacrylate, dl-Camphorquinone, Water	BQ0055	Kuraray Noritake, Okayama, Japan	After the nHAp_PA, apply the primer with an applicator and leave it in place for 20 s. Then, dry for 5 s with mild air flow.
	Bond: 10-MDP, HEMA, Bis-GMA, Hydrophobic aliphatic dimethacrylate, dl-Camphorquinone, Initiators, Accelerators, Silanated colloidal silica	BJ0081		After the primer, apply the adhesive and light-cure for 10 s.
Brilliant EverGlow	Methacrylates, dental glass, amorphous silica, zinc oxide	J39958	Coltene, Altstatten, Switzerland	After bonding procedures, apply 2 mm layers of composite and light-cure each increment layer for 20 s.

*Abbreviations:* 10-MDP, 10-methacryloyloxydecyl dihydrogen phosphate; HEMA, 2-hydroxyethyl methacrylate; Bis-GMA, bisphenol A diglycidylmethacrylate

### Microtensile bond strength (µTBS) test

Ten bonded teeth per group were sectioned in the “x” and “y” directions perpendicular to the bonding interface to obtain sticks with a cross-section area of approximately 1 mm^2^ using a diamond cut-off wheel (MOD 13, Struers, Ballerup, Denmark) on an Accutom 50 cutting machine (Struers, Ballerup, Denmark) under water cooling. The cross-sectional area of the bonded interface of each stick was measured with a digital caliper (IP67, Mahr GmbH, Gottingen, Germany). Sticks from each tooth were randomly divided into two subgroups: 24 h (T0) or 6 months aging (T6) at 37 °C, renewing the distilled water weekly, before µTBS measurements. The sticks were fixed in a specific microtension test holder using cyanoacrylate (Cofan Cyanoacrylate, Cofan la Mancha, Ciudad Real, Spain). The microtensile strength was determined with an Instron 3345 machine (Instron Co., Massachusetts, USA) with a 500 N load cell at a crosshead speed of 1 mm/min until fracture. The µTBS was expressed in MPa.

Failures were analyzed using an SZ-TP stereomicroscope (Olympus, Tokyo, Japan) at 50X objective to determine the fracture mode and classified as cohesive in dentin (CD), cohesive in composite (CC), mixed (M), and adhesive (A). One fractured specimen per group was randomly selected for representative interfacial images using Scanning Electron Microscopy (SEM; Phenom XL G2 Desktop SEM, Thermo Fisher Scientific, Waltham, USA) at 10 kV. Before SEM analysis, all specimens were processed using a common processing protocol for SEM: fixation, gradual alcohol dehydration, and coating with carbon.

### Gelatin zymography

Gelatin zymography was performed according to the method employed by Mazzoni et al., 2013 [24]. Briefly, enamel, cement, and pulp tissue were removed from 10 human healthy teeth extracted for orthodontic reasons. The teeth were further powdered with a Retsch mill (Model MM400, Retsch GmbH, Haan, Germany) and aliquots were made for five following groups: (i) negative control (mineralized); (ii) positive control demineralized with 10% phosphoric acid for 10 min at 4 °C; (iii) demineralized as previously described and then treated with nHAp_PA 2%; (iv) demineralized as previously described and then treated with nHAp_PA 6.5%; (v) demineralized as previously described and then treated with nHAp_PA 15%. The treatments with nHAp PA-containing solutions were performed for 30 min at 4 °C under constant agitation protected from the light. After washing and sonicating, the dentin powders were suspended in an extraction buffer (TRIS-HCl, CaCl_2_, NaCl, Triton X-100, NONIDET, ZnCl_2_, NaN_3_, Protease Inhibitor Tablets, EDTA-free, pH 6). overnight at 4 °C. Then, the specimens were sonicated for 10 min and centrifuged for 20 min. Supernatants were collected and concentrated using a Vivaspin centrifugal concentrator (10,000 kDa cut off; Vivaspin Sartorius Stedim Biotech, Goettingen, Germany) at 25 °C, 10,000 rpm, and when the amount of the concentrated liquid reached ≈100 µl, the total protein concentration in the dentin was determined by the Bradford assay using NanoDrop 2000c (Thermo Fisher Scientific, Wilmington, DE, USA). Aliquots of dentin protein were diluted with Laemmli sample buffer. Electrophoresis was performed under non-reducing conditions using 10% sodium dodecyl sulfate-polyacrylamide gel (SDS-PAGE) containing 1 mg/mL fluorescently labelled porcine gelatin. After electrophoresis, the gels were washed and incubated in a zymographic activation buffer (NaCl, CaCl_2_, TRIS-HCl, pH 8) for 48 h at 37 °C. Proteolytic activity was evaluated and recorded with a long-wave ultraviolet light scanner (Chemi-Doc Universal Hood, Bio-Rad).

### In situ zymography of resin-dentin interfaces

Two bonded sticks from 5 teeth per group prepared for µTBS were randomly selected, at T0 as well as at T6. In situ zymography was performed according to the protocol reported by Mazzoni et al., 2012 [25], using self-quenched fluorescein-conjugated gelatin as the MMP substrate. Briefly, the specimens were glued to a microscope slide, progressively polished with wet silicon carbide paper (grit sizes: 600, 1,200, 4,000) until ~ 50 μm thickness, coated with fluorescein-conjugated gelatin mixture (E-12,055; Molecular Probes, Eugene, OR, USA), and covered with a coverslip. The specimens were placed in a humid chamber and kept overnight in the dark at 37 °C. After incubation, the microscope slides were examined using a laser scanning confocal microscope (Leica SP8, Leica Microsystems GmbH, Wetzlar, Germany; excitation/emission wavelength: 488/530 nm). Two z-stack images were made for each sample by a researcher who was unaware of the designated groups. The same laser power and gain settings were used at T0 and T6. Quantification of the integrated density of the fluorescence signal was performed at the same level for each image via image analysis software (ImageJ; National Institutes of Health, Maryland, USA). A standardized rectangular selected area was used for all images. Image acquisition and quantification procedures are explained in detail elsewhere [26]. Differences in the level of fluorescence among the tested groups expressed as Integrated density (pixels/µm^2^) were statistically analyzed.

### Statistical analysis

Statistical analysis was conducted via IBM SPSS 23.0 (SPSS Inc., Chicago, USA). The µTBS data analysis was performed using the tooth as a statistical unit (*n* = 10 teeth) by averaging the bond strength of the sticks of each tooth per aging time. The normal distribution and the equal variance of µTBS and in situ zymography data were confirmed with Shapiro-Wilk and Levene tests, respectively. Two-Way Analyses of Variance (ANOVA; variables: treatment method and time of ageing) and Tukey’s post hoc test were used to analyze the data. Differences between the aging times of each group were investigated using paired *t*-tests. The significance level was set at *p* < 0.05.

## Results

### Microtensile bond strength (µTBS) test

**Fig. 2:**
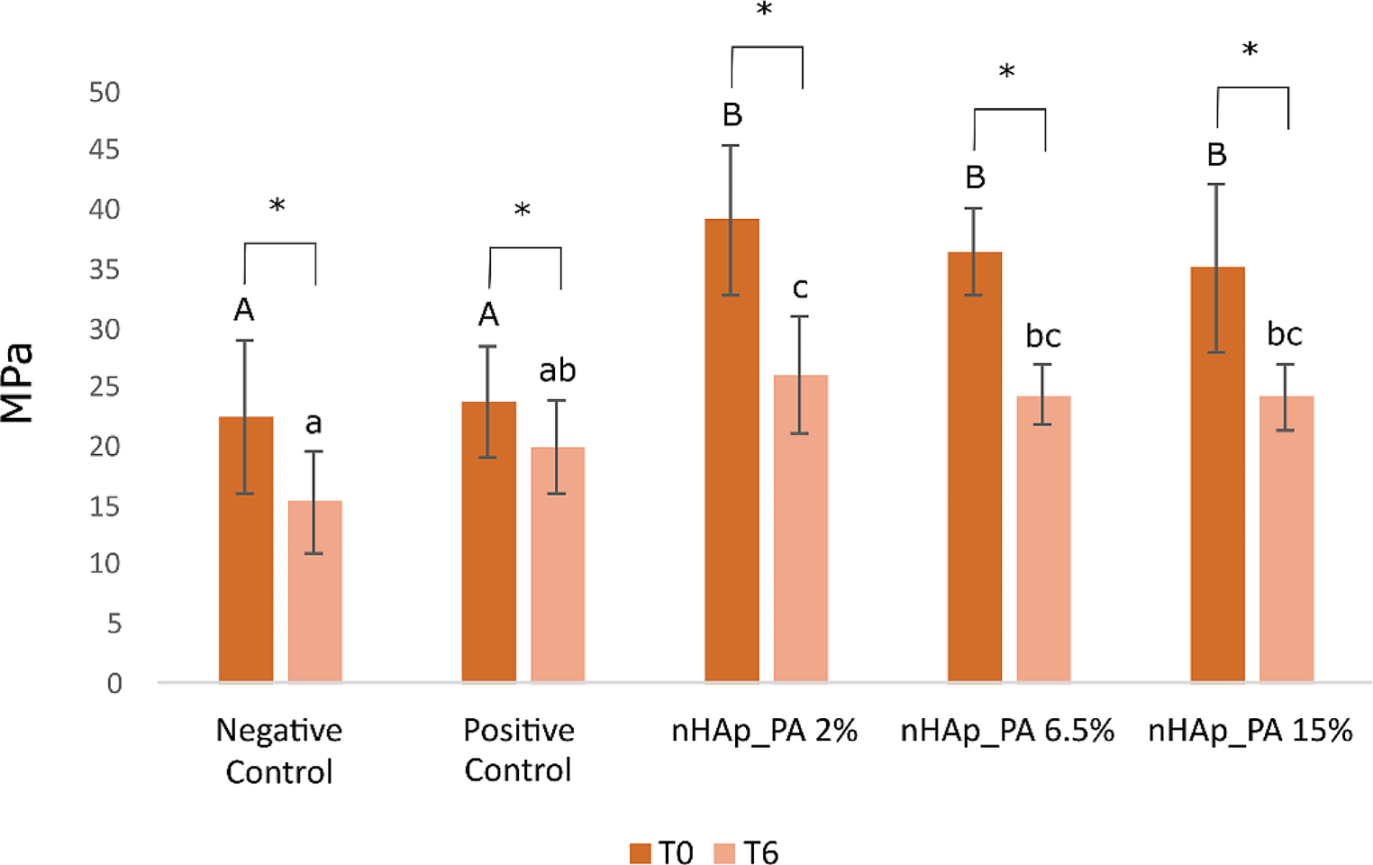
Graph summarizing the microtensile bond strength test values at 24 h (T0) and after 6 months of artificial aging (T6). Different capital letters indicate significant differences between groups at T0. Different lower-case letters indicate significant differences between groups at T6. Asterisks indicate significant differences between the times studied in each group. Significance *p* < 0.05

The main results from the microtensile bond strength tests (µTBS) are presented in Fig. 2. Two-way ANOVA analysis showed a significant interaction between aging time *versus* treatment method (*p* = 0.02). At T0, all treated samples showed higher µTBS than control samples (*p* ≤ 0.001), with no differences between them. After 6 months aging, the group treated with nHAp_PA 2% had a higher µTBS compared to all the other groups, and specimens from the negative control had a lower µTBS than the other groups with values changing in the following order: nHAp_PA 2% ≥ nHAp_PA 6.5% = nHAp_PA 15% ≥ positive control ≥ negative control (*p* ≤ 0.007). Further, paired t-tests showed that all groups had decreased values at T6 compared to T0 (*p* ≤ 0.008). There was an average of 1 premature failure in each group that was not included in the statistical analyses.

**Fig. 3:**
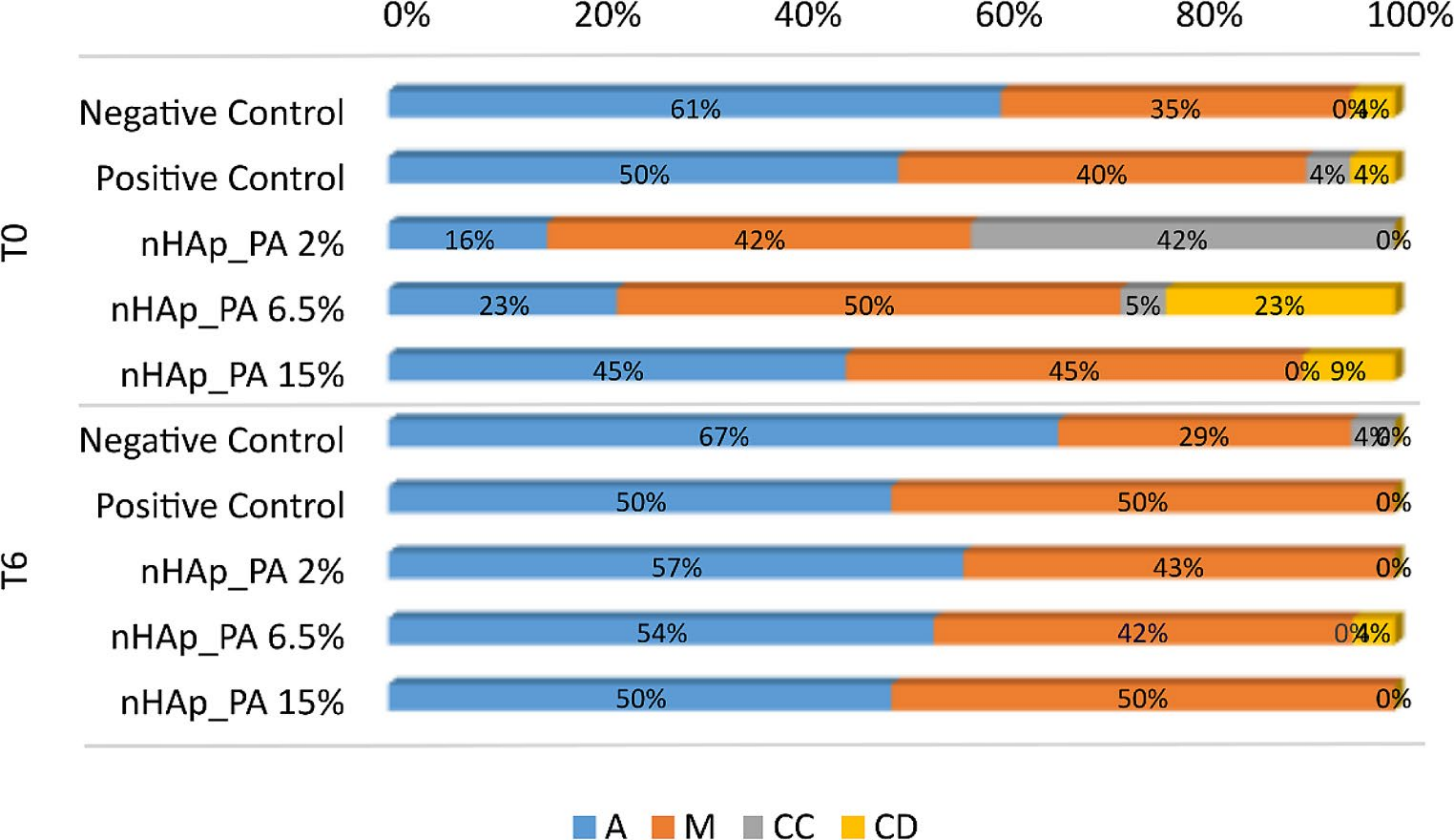
Percentages of the failures modes: adhesive (**A**), mixed (M), cohesive in dentin (CD), and cohesive in composite (CC) at baseline (T0) and 6 months aging (T6)

**Fig. 4:**
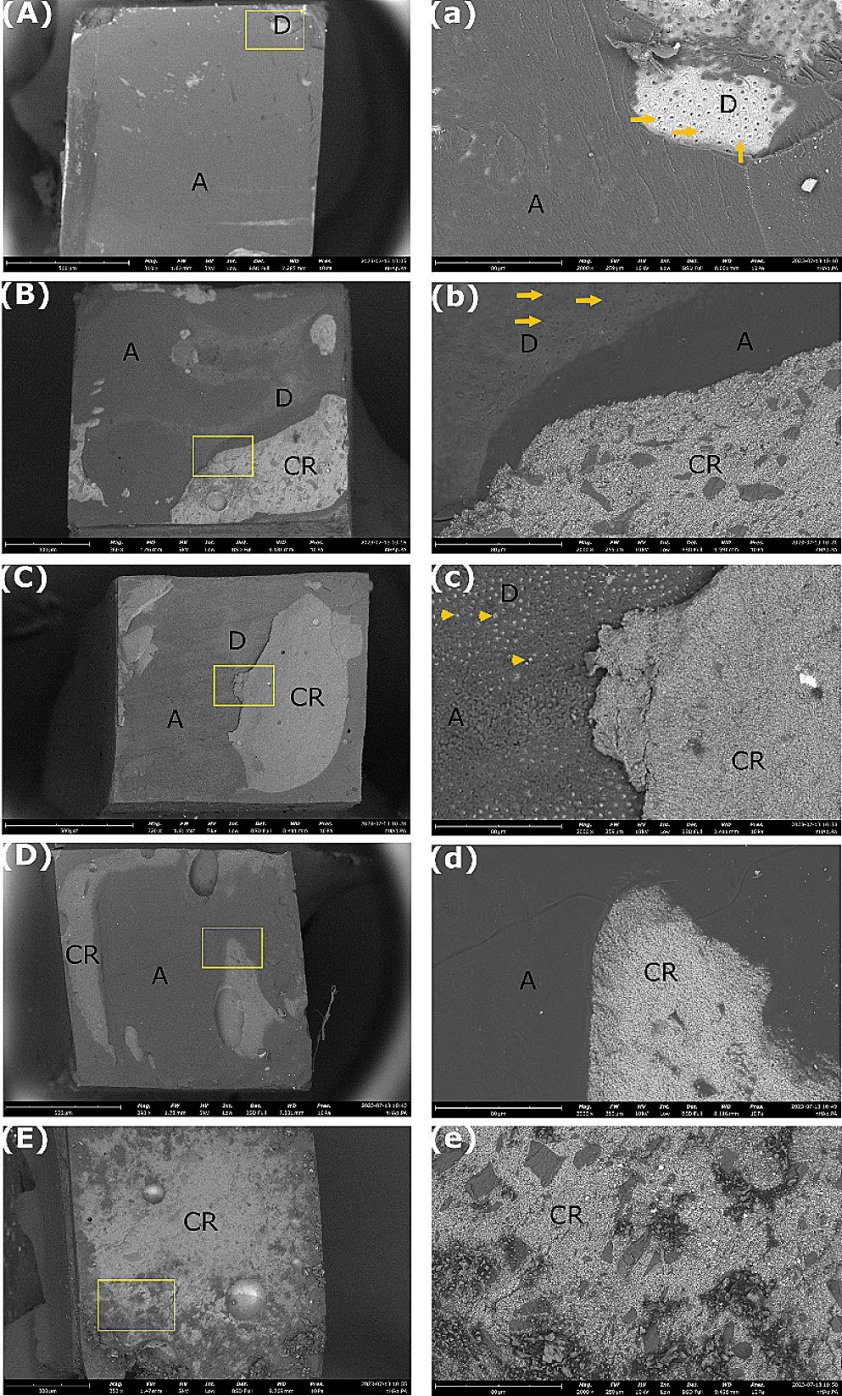
Representative SEM images of the fractured dentin surfaces at T0 (baseline). (**A** – **E**) Low magnification (500 X, bars: 500 μm) and (**a** – **e**) high magnification (2000 X, bars: 80 μm) of the areas limited by the yellow section in (**A** – **E**). (**A**) Negative control group with adhesive failure. (**a**) Presence of an adhesive layer and open dentin tubules. (**B**) Positive control group with mixed failure. (**b**) Presence of open dentin tubules, adhesive layer, and composite resin. (**C**) nHAp_PA 2% with mixed failure. (**c**) Presence of dentin tubules sealed by resin tags, adhesive layer, and composite resin. (**D**) nHAp_PA 6.5% with mixed failure. (**d**) Presence of adhesive layer and composite resin. (**E**) nHAp_PA 15% with cohesive failure in composite resin. (**e**) Presence of resin composite on the entire surface. Arrows: open dentin tubules. Arrowheads: dentin tubules sealed by resin tags. A: Adhesive. D: Dentin. CR: composite resin

**Fig. 5:**
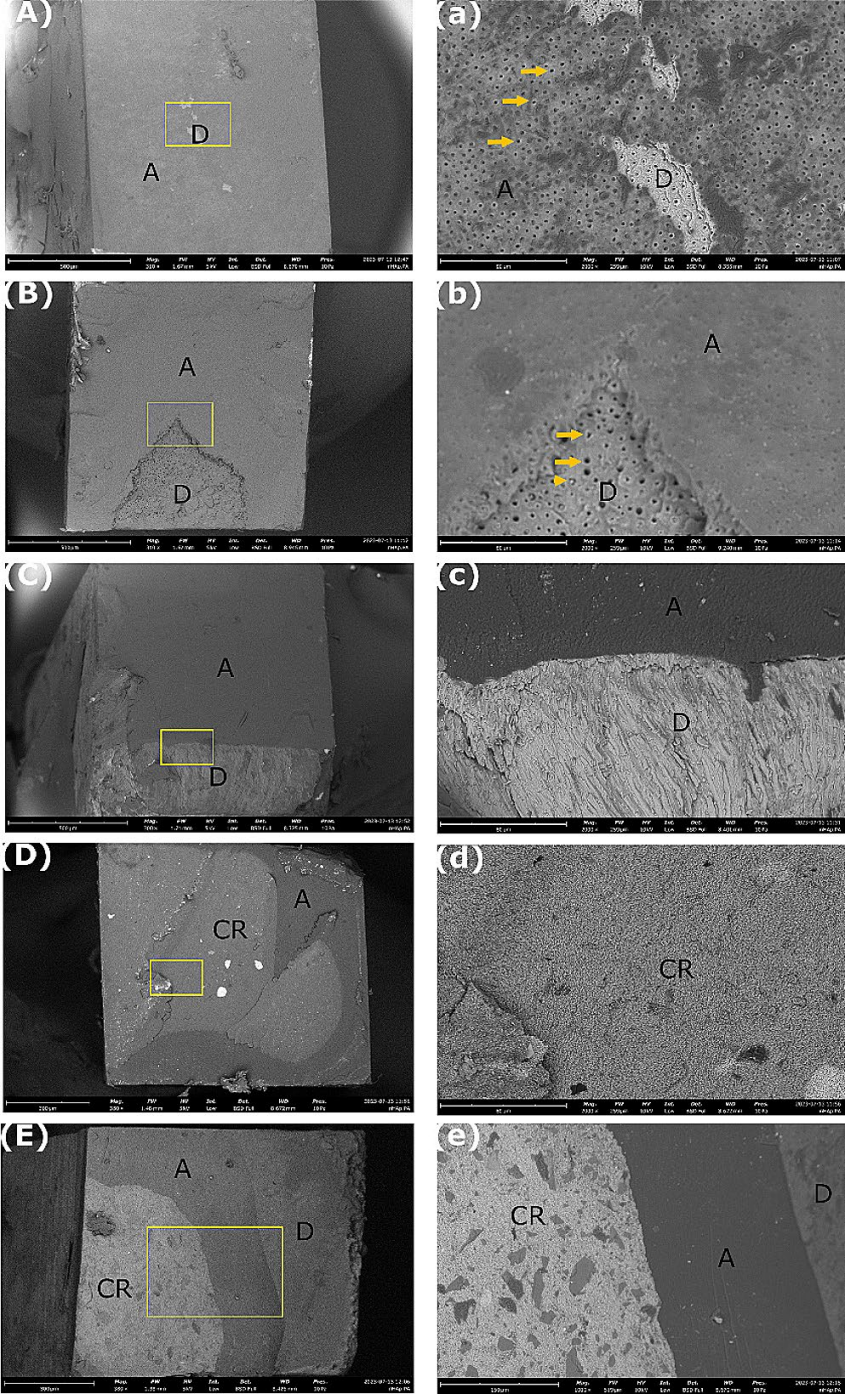
Representative SEM images of the fractured dentin surfaces at T6 (6 months aging). (**A** – **E**) Low magnification (500 X, bars: 500 μm) and (**a** – **e**) high magnification (2000 X, bars: 80 μm) of the areas limited by the yellow section in (**A** – **E**). (**A**) Negative control group with adhesive failure. (**a**) Presence of an adhesive layer and several open dentin tubules. (**B**) Positive control group with adhesive failure. (**b**) Presence of mostly open dentin tubules and an adhesive layer. (**C**) nHAp_PA 2% with adhesive failure. (**c**) Presence of an adhesive layer and a fracture in dentin. (**D**) nHAp_PA 6.5% with mixed failure. (**d**) Presence of the composite resin. (**E**) nHAp_PA 15% with mixed fracture. (**e**) Presence of dentin, adhesive layer, and composite resin. Arrows: open dentin tubules. Arrowhead: dentin tubules sealed by resin tags. A: Adhesive. D: Dentin. CR: composite resin

The type of fracture patterns in dentin specimens from the different experimental groups were evaluated by their frequency distribution and the main results are summarized in Fig. 3. Adhesive and mixed failures predominated in all groups at both T0 and T6. The prevalence of mixed failures was higher in the treated groups, while adhesive failures were prevalent in the control groups at baseline. It is worth noting that nHAp_PA 2% treated dentin specimens showed 42% of resin composite fractures at T0. The incidence of adhesive failures increased after aging, especially in the experimental groups compared to controls. At 6 months of aging, the majority of the fracture mode was adhesive in all groups. Figures 4 and 5 show representative SEM images of the fractured dentin surfaces at T0 and T6 aging time of all studied groups. These images showed the presence of open dentin tubules in control groups at both T0 and T6 and dentin tubules sealed by resin tags in the nHAp_PA 2% group at T0.

### Gelatin zymography

**Fig. 6:**
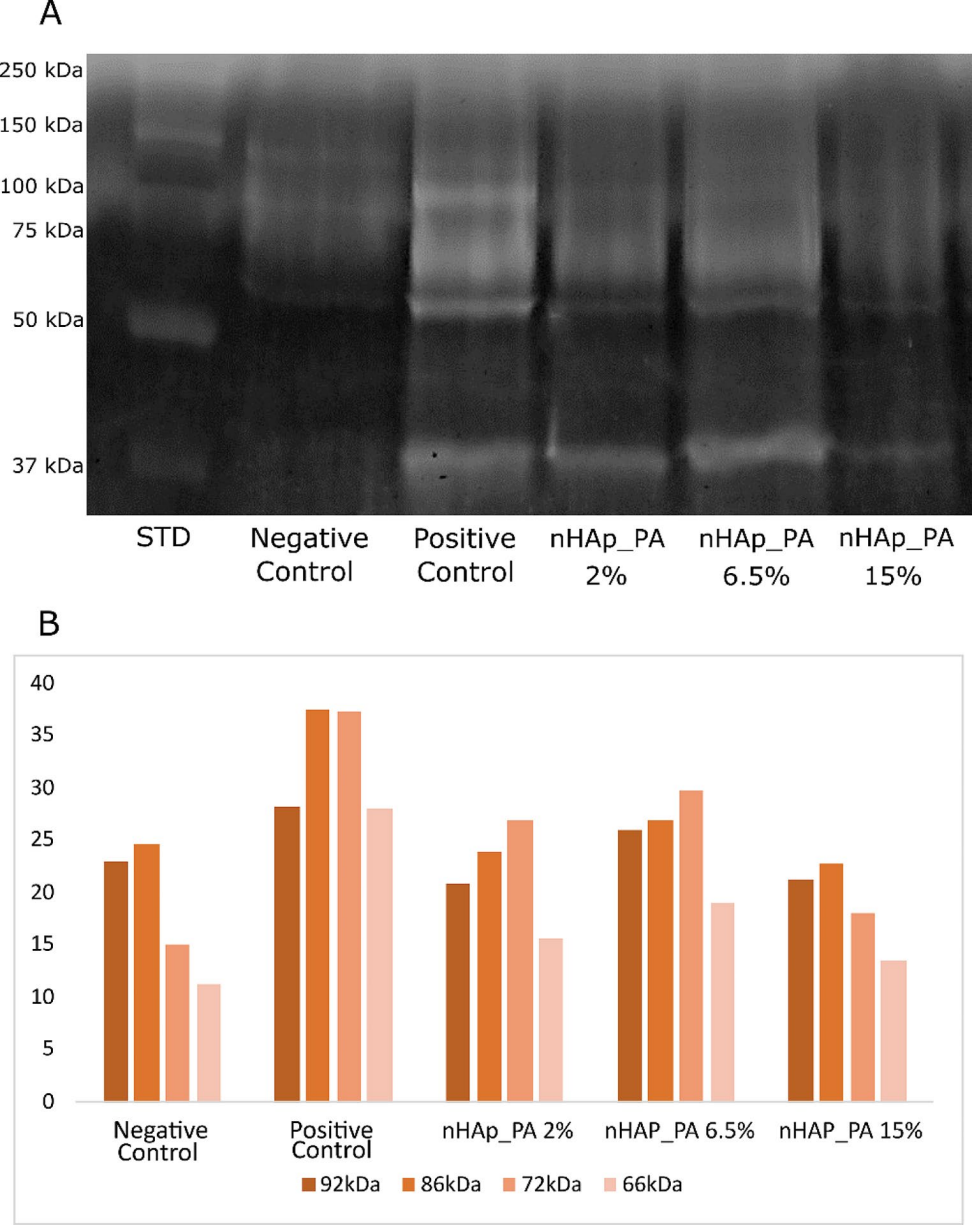
(**A**) Gelatin zymography analysis of standard (STD), negative control, positive control, and treated groups. (**B**) Quantification of the band pixels from the gelatin zymography analysis

Figure 6 shows the qualitative and quantitative data of gelatin zymography. Positive control (i.e., demineralized dentin) showed the highest enzymatic activity of the pro- and active forms of MMP-2 (72 kDa and 66 kDa, respectively) and MMP-9 (92 kDa and 86 kDa, respectively), while negative control (i.e., mineralized dentin) showed the lowest activity of both MMPs. In addition, all nHAp_PA-treated groups demonstrated a reduction in enzymatic activity compared to the positive control group presenting almost the same level of gelatinolytic activity as the negative control group.

### In situ zymography of resin-dentin interfaces

**Fig. 7:**
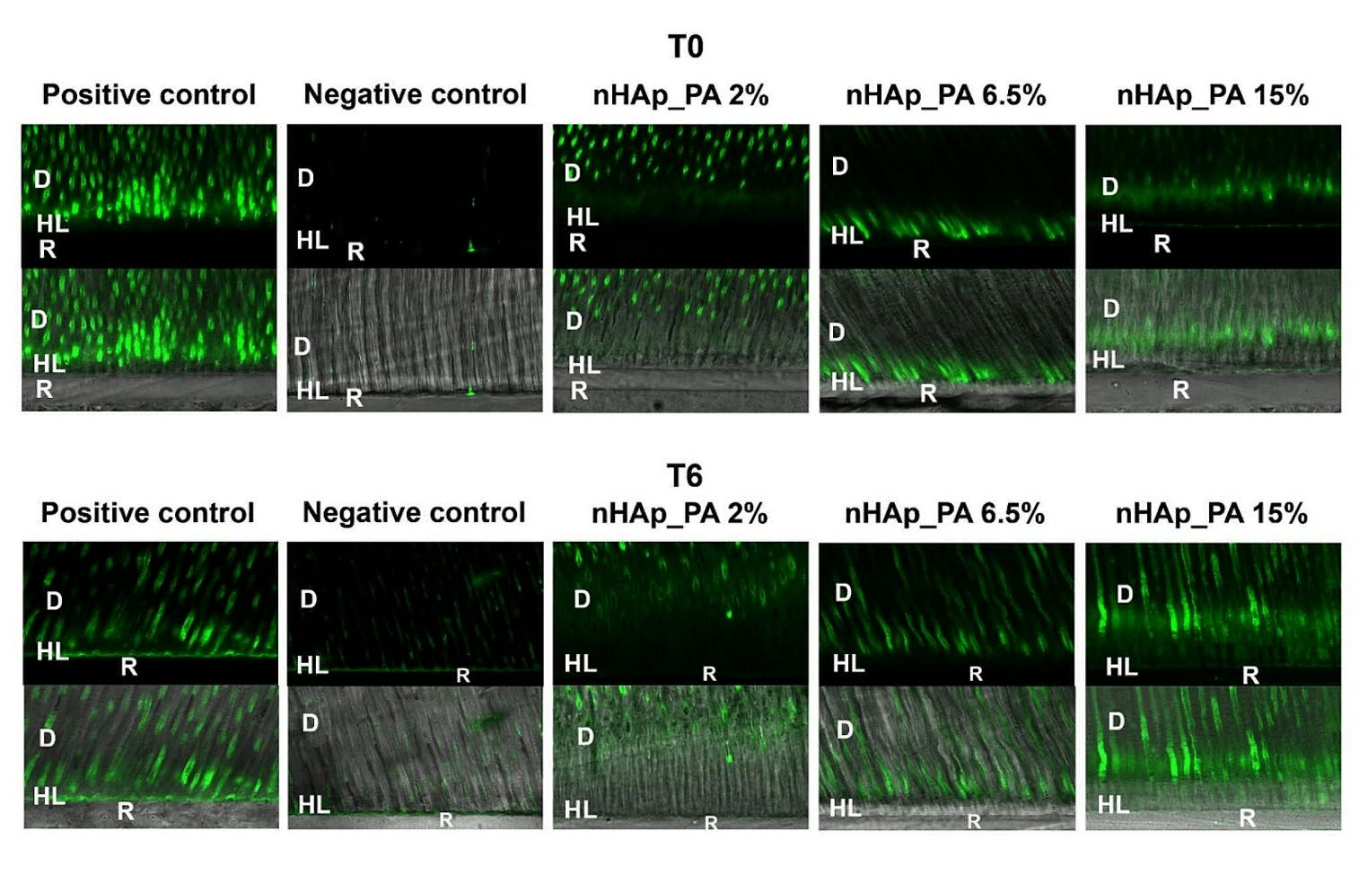
Resin-dentin interfaces incubated with quenched fluorescein-labeled gelatin at T0 and T6 months. Upper image in each group was acquired in the green channel showing fluorescence in dentinal tubules and within the HL of the tested groups. The lower image in each group was obtained by merging the differential interference contrast (DIC) image (showing optical density of the resin-dentin interface) and the image acquired in the green channel. Abbreviations: D - dentin; HL – hybrid layer; R – resin composite; T0–24 h aging; T6–6 months aging

**Fig. 8:**
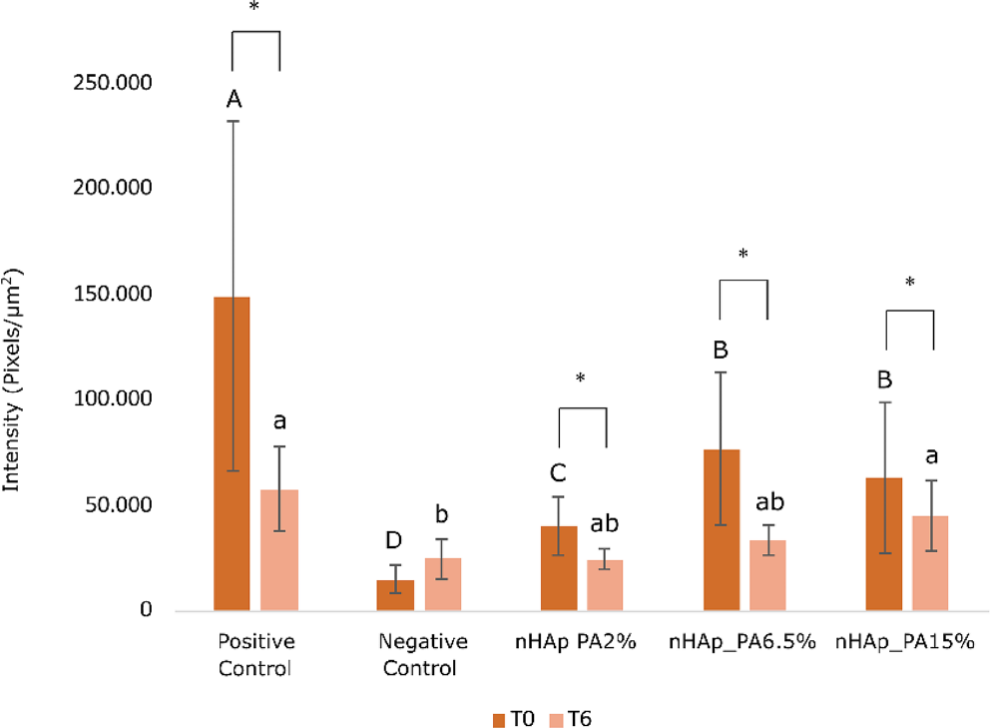
Quantification of the gelatinolytic activity within the resin-dentin interfaces of the tested groups. Different capital letters indicate significant differences between groups at T0. Different lower-case letters indicate significant differences between groups at T6. Asterisks indicate significant differences between the times studied in each group. Significance *p* < 0.05

The enzymatic activity at the resin-dentin interface was analyzed in situ by confocal microscopy. Figure 7 shows representative images of the resin-dentin interface for all experimental groups. The presence of gelatinolytic activity is evidenced by the fluorescence signal which is mainly distributed in tubules being especially intense in the positive control. Figure 8 shows the quantitative results of the gelatinolytic activity levels within the resin-dentin interfaces for all the tested groups. It can be noted that the main effects (treatment and aging), as well as their interaction, significantly influenced the level of fluorescence within the dentin observed in the different groups (*p* < 0.001).

Considering the investigated treatments at T0, positive control demonstrated significantly higher enzymatic activity compared to other tested groups (*p* < 0.001). Pretreatment with nHAp_PA lowered dentinal enzymatic activity compared to positive control. However, it was not able to fully inhibit the enzymatic activity and reduce it to the level of negative control, regardless of the concentration of nanoparticles in the pretreatment primer (positive control > nHAp_PA 15% = nHAp_PA 6.5% > nHAp_PA 2% > negative control). After aging (T6), the gelatinolytic activity in the positive control group remained higher than in the negative control group, nHAp_PA 6.5% and nHAp_PA 2% (*p* ≤ 0.009), which was not different from nHAp_PA 15% (*p* = 0.326). nHAp_PA 15% demonstrated significantly higher gelatinolytic activity compared to the negative control group (*p* = 0.035), whereas there were no significant differences between the other investigated groups. On the other hand, the enzymatic activity decreased significantly after 6 months of artificial aging (*p* < 0.05) in all groups except in the negative control group (*p* ≤ 0.031), where the same (low) fluorescence level was maintained.

## Discussion

Despite the rapid development of adhesive dentistry in recent decades, strategies to prevent dentin bonding degradation and promote the durability of the adhesive interface are still necessary. In the present study, the effects of proanthocyanidin-functionalized hydroxyapatite (nHAp_PA) nanoparticles used as a pretreatment primer on the longevity of the adhesive-dentin interface were investigated. Our results confirmed that the application of nHAp_PA to pH-cycled dentin improved dentin bond strength and reduced endogenous dentinal enzymatic activities at baseline and after 6 months. Furthermore, nHAp_PA 2% demonstrated the most promising results over the analyses studied. Therefore, the tested null hypotheses were rejected because there was a difference between the experimental groups in microtensile bond strength and MMPs activity. Moreover, at T6, both bond strength values and the endogenous enzymatic activity decreased for all groups compared to T0, requiring the rejection of the third null hypothesis.

The E&R strategy eliminates the smear layer by completely demineralizing the surface of the dentin; however, the monomers do not infiltrate deeper layers, generating areas of denuded collagen [4, 27]. In the SE technique, the smear layer is maintained as a substrate for adhesion, providing protection to the collagen and the remaining crystals, which can be an important source of calcium for bonding with the monomers [4, 28]. Although both the E&R and SE techniques increase the enzymatic activity, etch-and-rinse adhesives generally exhibit higher activity than self-etch adhesives [24]. This is related to the inability of E&R adhesive resins to penetrate the full depth of the etching, leaving the demineralized dentin matrix partially uncovered [24]. The localization of enzyme activity was in fact correlated with the non-infiltrated denuded collagen areas generated by the etch-and-rinse adhesives [29]. As our aim was to evaluate different dentin treatments, we decided to use the two-step self-etch mode because this is the preferred bonding protocol for this substrate [28, 30]. Furthermore, the nHAp_PA concentrations determined in this study were based on commonly used concentrations in previous reports [31–35]. Moreover, we tested the nanoparticles on demineralized dentin created by a pH-cycling process. This method is more appropriate for simulating a substrate comparable to the caries-affected dentin layer [23].

Proanthocyanidin forms strong insoluble bonds between its phenolic hydroxyl group and the carbonyl amide group of dentin collagen [9]. This interaction between collagen and PA can increase the hardness of demineralized dentin, maintaining the integrity of the complex structure of collagen, facilitating the interdiffusion of monomers, favoring remineralization, and inhibiting collagenase activity [36, 37]. Systematic reviews and meta-analyses concluded that due to the properties mentioned above, the application of collagen cross-linkers as a pretreatment increased the immediate and long-term dentin bond strength [36–38]. This finding is in accordance with our results, as the treated groups showed higher µTBS than the controls at both T0 and T6 (Fig. 2). At 6 months of aging, no significant differences were observed between the positive control, nHAp_PA 6.5%, and nHAp_PA 15%. However, the nHAp_PA 2% showed higher µTBS compared to control groups. Therefore, our findings are consistent with those of other studies, in which the more concentrated the PA pretreatment, the lower the bond strength [39]. This could be because PA donates atoms to free radicals in direct proportion to the PA concentration, inhibiting the initiation and propagation of adhesive polymerization [39]. Moreover, the addition of nHAp at higher concentrations has a negative effect on bond strength [40]. This may be due to the fact that at low concentrations, nHAp can infiltrate dentinal tubules, thereby increasing mechanical properties, whereas at higher concentrations, particles agglomerate on the surface and reduce adhesive penetration into the dentin [41, 42]. Nevertheless, some reports have suggested that dentinal tubule occlusion by mineral deposition not only facilitates remineralization but also reduces interface degradation and increases immediate and long-term bond strength [43, 44]. According to our findings, nHAp did not impair bond strength and could potentially serve as a calcium- and phosphate-releasing source. Furthermore, the higher µTBS of the treated groups can be attributed to the cumulative effect of PA performance combined with nHAp, as nHAp also can interact with dentin apatite, strengthening and stabilizing the dentin matrix [45]. Moreover, self-etch adhesive systems have functional monomers that chemically interact with dentin hydroxyapatite [28]. The Clearfil Se Bond 2 self-etch adhesive used in the present study contains 10-methacryloxydecil dihydrogen phosphate (10-MDP) monomer, which forms a strong chemical bond with dentin hydroxyapatite calcium, resulting in the formation of a new calcium phosphate [28]. Additionally, the application of nHAp as a pretreatment on dentin provides Ca^2+^ sites for chemical reactions with 10-MDP, thereby establishing a chemical-mechanical bond [46, 47]. This potential interaction between nHAp and the adhesive system may also be another factor that explains the increased bond strength of the treated groups.

The in vitro pH-cycling model provides alternating periods of demineralization and remineralization, producing a demineralized dentin surface similar to caries-affected dentin [23]. However, the breakdown of dentin collagen cannot be reproduced by pH-cycling; therefore, this method provides demineralized dentin with intact collagen [23, 48]. It is interesting to note that there was no difference in the microtensile strength between the negative and positive controls at any of the time points studied. Considering the role of collagen in dental adhesion, post-pH-cycled intact collagen may have generated microtensile strength values similar to those of the negative control group.

The frequency distribution of fracture patterns (Fig. 3) showed that at T0, most fracture modes in the treated groups were mixed, whereas in the control groups, they were adhesive. At T6, all groups showed the majority of adhesive failures. Interestingly, the nHAp_PA 2% group had 42% resin composite fractures at T0 (Fig. 3). This could indicate that the resin-dentin bond strength promoted by this pretreatment exceeded the cohesive strength within the composite build-up. Additionally, SEM images showed resin tags inside the dentinal tubules in this group (Fig. 4c), whereas the control groups had open tubules at T0 (Fig. 4a and b). At T6, the control groups also exhibited mostly open tubules (Fig. 5a and b). Nevertheless, higher magnifications should be used to confirm these findings.

When activated by acid agents during the adhesive protocol, MMPs and CTs detected at the bottom of the HL degrade the water-rich type I collagen that was not infiltrated with resin monomers [3]. Therefore, the use of bioactive crosslinking pretreatments to inactivate MMPs and prevent degradation of the adhesive interface is a valid strategy to prolong the longevity of the bonded restoration [49, 50]. Their mode of action is two-fold. On one hand, they stabilize and reinforce the dentin matrix, while on the other hand, they are nonspecific inhibitors of MMPs due to their ability to induce conformational changes in the enzymes themselves and, simultaneously, mask the enzyme’s cleavage sites on the collagen molecule [50, 51]. It is known that the phenolic hydroxyl group of PA interacts with the carbonyl protein amide of dentin collagen via covalent, ionic, hydrogen, and hydrophobic bonds, forming strong and permanent crosslinks [9]. Furthermore, the large galloyl moiety of PA offers more hydroxyl groups for crosslinking, leading to a better performance. [9]. In the current study, the detection of MMP activity was performed by gelatin zymography and in situ zymography. Gelatin zymography of dentin powder revealed higher enzymatic activity in the positive control group, while the negative control group showed the lowest activity (Fig. 6). Moreover, the use of nHAp_PA as a pretreatment decreased the enzymatic activity of both the pro-form and active forms of MMP-2 and MMP-9. Also, treatment groups presented almost the same intensity of enzymatic activity as the negative control group, confirming the MMP inactivation potential of dentin pretreatment with nHAp_PA. In situ zymographic analysis of dentin-resin interfaces corroborated the effectiveness of pretreatments for inhibiting endogenous dentinal gelatinolytic activity (Figs. 7 and 8). The results showed that the pH-cycling process before application of the adhesive system induced the endogenous dentinal enzymatic activity because the positive control presented the highest activity. Moreover, after 6 months of aging, nHAp_PA 2% and nHAp_PA 6.5% were able to decrease enzymatic activity at the resin-dentin interface to the level of the negative control. Interestingly, all groups (apart from the negative control) demonstrated a decrease in enzymatic activity after aging, although it would be expected that the activity would increase after aging. This could be explained by the fact that the specimens were aged in distilled water, and not in the artificial saliva rich in minerals necessary for MMP function. A decrease in gelatinolytic activity assessed using in situ zymography in water-aged specimens has been reported previously [52]. It is important to highlight the strengths and weaknesses of the zymographic assays used in the present study. Gelatin zymography enables to precisely demonstrate the expression and activity of endogenous dentinal MMP-2 and MMP-9, gelatinases that are paramount in HL degradation as well as caries development [2, 53–57]. However, it does not simulate actual clinical procedures due to the fact that dentin powder, which has a much larger surface compared to a dentin block, is used as a substrate, influencing the interaction between the materials and dentin. On the other hand, in situ zymography perfectly mimics the clinical protocol, but the enzymatic activity presented by the fluorescence cannot be attributed to any particular enzyme, and is a combination of the activity of all gelatinolytic enzymes present in dentin, such as MMP-2, MMP-9, CT, etc. Nevertheless, these two tests combined provide a valuable insight in the endogenous enzymatic activity of dentin when treated with various dental materials [2, 7, 53–59].

Thus, the findings of the present study indicate that nHAp_PA can inactivate MMPs for a medium-long period and increase bond strength. The results of this study complement those of previous in which the application of nHAp_PA remineralized and strengthened demineralized dentin matrix [21]. Our findings validate the beneficial effects of pretreating dentin for 1 min with nHAp_PA on µTBS and the inactivation of MMP-9 and MMP-2. Moreover, according to the microtensile strength at 6 months and in situ zymography results, the nHAp_PA 2% pretreatment was found to have the best performance. Further laboratory studies with longer evaluation times are warranted, as are clinical trials to validate these benefits in vivo.

## Conclusion

The application of a pretreatment primer containing proanthocyanidin-functionalized hydroxyapatite nanoparticles prior to a self-etch adhesive procedure increases bond strength after 6 months of artificial aging with inhibition of host-derived enzymatic activity. The primer containing 2% nanoparticles appeared to be the most efficient treatment in terms of bonding and MMP inhibitory properties.

## Data Availability

No datasets were generated or analysed during the current study.
